# Supplementary material for: Characterization of the Evolutionary Pressure on Anisodus tanguticus Maxim. with Complete Chloroplast Genome Sequence
Source: Genes (Basel). 2022 Nov 15;13(11):2125. doi: 10.3390/genes13112125 (PMC9690199; doi:10.3390/genes13112125)
Supplement: Supplementary file 1 [file genes-13-02125-s001.zip › Table S1.pdf]

Table S1 The codon usage and codon-anoticodon recognition pattern for the *A. tanguticus* cp genome.

Amino acid	Codon	Number	RSCU	tRNA	Amino acid	Codon	Number	RSCU	tRNA
Ter	UAA	46	1.5861		Met	AUG	631	1.9936	trnM-CAU
Ter	UAG	23	0.7932		Met	GUG	2	0.0064	
Ter	UGA	18	0.6207		Met	UUG	0	0	
Ala	GCA	397	1.1272	trnA-UGC	Asn	AAC	312	0.4674	trnN-GUU
Ala	GCC	248	0.704		Asn	AAU	1023	1.5326	
Ala	GCG	133	0.3776		Pro	CCA	333	1.1756	trnP-UGG
Ala	GCU	631	1.7912		Pro	CCC	201	0.7096	
Cys	UGC	83	0.5442	trnC-GCA	Pro	CCG	156	0.5508	
Cys	UGU	222	1.4558		Pro	CCU	443	1.564	
Asp	GAC	220	0.4048	trnD-GUC	Gln	CAA	722	1.4994	trnQ-UUG
Asp	GAU	867	1.5952		Gln	CAG	241	0.5006	
Glu	GAA	1060	1.495	trnE-UUC	Arg	AGA	491	1.8138	trnR-UCU
Glu	GAG	358	0.505		Arg	AGG	175	0.6468	
Phe	UUC	541	0.7082	trnF-GAA	Arg	CGA	390	1.4406	
Phe	UUU	987	1.2918		Arg	CGC	100	0.3696	
Gly	GGA	735	1.5928	trnG-UCC	Arg	CGG	120	0.4434	
Gly	GGC	204	0.442	trnG-GCC	Arg	CGU	348	1.2858	trnR-ACG
Gly	GGG	323	0.7		Ser	AGC	122	0.3486	trnS-GCU
Gly	GGU	584	1.2656		Ser	AGU	423	1.2078	
His	CAC	145	0.464	trnH-GUG	Ser	UCA	424	1.2108	trnS-UGA
His	CAU	480	1.536		Ser	UCC	337	0.9624	trnS-GGA
Ile	AUA	700	0.9279		Ser	UCG	193	0.5514	
Ile	AUC	452	0.5991	trnI-GAU	Ser	UCU	602	1.719	
Ile	AUU	1111	1.4727	trnI-CAU	Thr	ACA	407	1.1908	trnT-UGU
Lys	AAA	1075	1.4828	trnK-UUU	Thr	ACC	280	0.8192	trnT-GGU
Lys	AAG	375	0.5172		Thr	ACG	154	0.4508	
Leu	CUA	374	0.7824	trnL-UAG	Thr	ACU	526	1.5392	
Leu	CUC	212	0.4434		Val	GUA	543	1.4848	trnV-UAC
Leu	CUG	201	0.4206		Val	GUC	187	0.5112	trnV-GAC
Leu	CUU	622	1.302		Val	GUG	201	0.5496	
Leu	UUA	879	1.8396	trnL-UAA	Val	GUU	532	1.4544	
Leu	UUG	579	1.212	trnL-CAA	Tyr	UAC	190	0.3866	
Trp	UGG	483	1	trnW-CCA	Tyr	UAU	793	1.6134	trnY-GUA

RSCU: Relative synonymous Codon Usage; Ter : terminal codon
