# Supplementary material for: Characterization of the Evolutionary Pressure on Anisodus tanguticus Maxim. with Complete Chloroplast Genome Sequence
Source: Genes (Basel). 2022 Nov 15;13(11):2125. doi: 10.3390/genes13112125 (PMC9690199; doi:10.3390/genes13112125)
Supplement: Supplementary file 1 [file genes-13-02125-s001.zip › Table S2.pdf]

Table S2a Comparison of repeat sequences on *A.tanguticus* and five related species.

Species	type	30-39bp	40-49bp	50-59bp	>60bp
	F	17	1	0	0
N.tabacum	P	13	2	1	1
	C	0	0	0	0
	R	1	0	0	0
	F	12	1	0	0
D.stramonium	P	10	1	1	1
	C	0	0	0	0
	R	0	0	0	0
	F	14	7	2	0
H.niger	P	11	7	3	1
	C	0	0	0	0
	R	0	0	0	0
	F	16	2	0	0
A.tanguticus	P	13	3	1	1
	C	0	0	0	0
	R	3	0	0	0
	F	19	3	1	0
P.tangutica	P	14	5	0	2
	C	0	0	0	0
	R	2	0	0	0
	F	15	4	0	0
S.parviflora	P	13	5	1	1
	C	0	0	0	0
	R	1	0	0	0

Note: F: forward repeats; C: complement repeat; R: reverse repeat; P: Palindromic repeat.

Table S2b Distribution of SSRs in the *A.tanguticus* cp genome.

SSR type	Unit	Times	Numbers of SSR
**P1**	**A**	**8**	**27**
	**A**	**9**	**5**
	**A**	**10**	**5**
	**A**	**11**	**3**
	**A**	**12**	**4**
	**A**	**13**	**1**
	**A**	**17**	**1**
	**C**	**8**	**2**
	**G**	**9**	**1**
	**T**	**8**	**27**
	**T**	**9**	**19**
	**T**	**10**	**8**
	**T**	**11**	**7**

	**T**	**12**		**2**	
	**T**	**13**		**1**	
	**T**	**14**		**2**	
	**T**	**15**		**1**	
	**T**	**16**		**2**	
	**T**	**17**		**2**	
**P2**	**AT**	**5**		**3**	
	**AT**	**6**		**1**	
	**TA**	**5**		**3**	
	**TA**	**6**		**1**	
**P3**	**AAC**	**3**		**2**	
	**AAG**	**3**		**4**	
	**AAT**	**3**		**5**	
	**AGA**	**3**		**3**	
	**ATA**	**3**		**3**	
	**ATC**	**3**		**1**	
	**ATG**	**3**		**1**	
	**ATT**	**3**		**3**	
	**CAA**	**3**		**1**	
	**CAG**	**3**		**2**	
	**CCT**	**3**		**1**	
	**CTC**	**3**		**1**	
	**CTG**	**3**		**1**	
	**CTT**	**3**		**2**	
	**GAA**	**3**		**2**	
	**GAG**	**3**		**1**	
	**GAT**	**3**		**1**	
	**GCT**	**3**		**1**	
	**GGA**		**3**		**1**
	**GTA**		**3**		**1**
	**GTG**		**3**		**1**
	**GTT**		**3**		**1**
	**TAA**		**3**		**5**
	**TAT**		**3**		**4**
	**TCG**		**3**		**1**
	**TCT**		**3**		**3**
	**TGA**		**3**		**1**
	**TGC**		**3**		**2**
	**TGG**		**3**		**1**
	**TTA**		**3**		**5**
	**TTA**		**4**		**1**
	**TTC**		**3**		**4**
	**TTC**		**4**		**1**
	**TTG**		**3**		**5**

P4	AAAC	3	1
	AAAT	3	1
	CAAA	3	1
	CTAT	3	1
	TTCT	3	1
	TTTA	3	1
	TTTG	3	1
P5	AATTG	3	1
