# Supplementary material for: Characterization of the Evolutionary Pressure on Anisodus tanguticus Maxim. with Complete Chloroplast Genome Sequence
Source: Genes (Basel). 2022 Nov 15;13(11):2125. doi: 10.3390/genes13112125 (PMC9690199; doi:10.3390/genes13112125)
Supplement: Supplementary file 1 [file genes-13-02125-s001.zip › Table S3.pdf]

Table S3 Comparison of nucleotide variability (Pi) among *A.tanguticus* and related species.

Region	Pi	Total Number of mutations	Region length
IR.gene1.trnN-GUU	0	0	72
IR.gene10.rps7	0	0	468
IR.gene11.ndhB	0.00149	6	1533
IR.gene12.trnL-CAA	0.00412	1	81
IR.gene13.ycf15	0.00156	1	264
IR.gene14.ycf2	0.00295	56	6894
IR.gene15.trnI-CAU	0	0	74
IR.gene16.rpl23	0.00236	2	282
IR.gene17.rpl2	0.00438	10	828
IR.gene2.trnR-ACG	0	0	74
IR.gene3.rrn5	0	0	121
IR.gene4.rrn4.5	0	0	103
IR.gene5.rrn23	0.00095	8	2810
IR.gene6.trnA-UGC	0	0	73
IR.gene7.trnI-GAU	0	0	77
IR.gene8.rrn16	0.00068	3	1500
IR.gene9.trnV-GAC	0	0	72
LSC.gene1.trnH-GUG	0	0	78
LSC.gene10.trnG-UCC	0	0	74
LSC.gene11.trnR-UCU	0.00463	1	72
LSC.gene12.atpA	0.01155	47	1524
LSC.gene13.atpF	0.00829	12	570
LSC.gene14.atpH	0.00623	4	246
LSC.gene15.atpI	0.00654	12	744
LSC.gene16.rps2	0.00591	12	711
LSC.gene17.rpoC2	0.01094	121	4182
LSC.gene18.rpoC1	0.00722	41	2067
LSC.gene19.rpoB	0.00421	37	3213
LSC.gene2.psbA	0.00477	14	1062
LSC.gene20.trnC-GCA	0	0	72
LSC.gene21.petN	0.00741	2	192
LSC.gene22.psbM	0	0	105
LSC.gene23.trnD-GUC	0.00721	1	74
LSC.gene24.trnY-GUA	0	0	84
LSC.gene25.trnE-UUC	0	0	73
LSC.gene26.trnT-GGU	0	0	72
LSC.gene27.psbD	0.00414	11	1062
LSC.gene28.psbC	0.00596	21	1422
LSC.gene29.trnS-UGA	0.00366	1	93
LSC.gene3.trnK-UUU	0	0	74
LSC.gene30.psbZ	0.00176	1	189
LSC.gene31.trnG-GCC	0	0	71

LSC.gene32.trnfM-CAU	0	0	74
LSC.gene33.rps14	0.00726	6	330
LSC.gene34.psaB	0.00511	32	2205
LSC.gene35.psaA	0.00571	33	2253
LSC.gene36.ycf3	0.00408	5	513
LSC.gene37.trnS-GGA	0.00383	1	87
LSC.gene38.rps4	0.00836	12	606
LSC.gene39.trnT-UGU	0	0	73
LSC.gene4.matK	0.01782	71	1530
LSC.gene40.trnL-UAA	0.00794	2	87
LSC.gene41.trnF-GAA	0.00457	1	73
LSC.gene42.ndhJ	0.00531	7	477
LSC.gene43.ndhK	0.0112	20	855
LSC.gene44.ndhC	0.01286	12	363
LSC.gene45.trnV-UAC	0	0	75
LSC.gene46.trnM-CAU	0	0	73
LSC.gene47.atpE	0.00829	8	402
LSC.gene48.atpB	0.00762	29	1497
LSC.gene49.rbcL	0.00735	28	1455
LSC.gene5.rps16	0.01731	12	267
LSC.gene50.accD	0.01449	60	1551
LSC.gene51.psaI	0.00901	3	111
LSC.gene52.ycf4	0.00456	7	555
LSC.gene53.cemA	0.01478	27	690
LSC.gene54.petA	0.01011	25	963
LSC.gene55.psbJ	0.00271	1	123
LSC.gene56.psbL	0.0057	2	117
LSC.gene57.psbF	0.00407	1	182
LSC.gene58.psbE	0.00265	2	252
LSC.gene59.petL	0.00903	2	96
LSC.gene6.trnQ-UUG	0	0	72
LSC.gene60.petG	0.00292	1	114
LSC.gene61.trnW-CCA	0	0	74
LSC.gene62.trnP-UGG	0.01502	2	74
LSC.gene63.psaJ	0.00741	3	198
LSC.gene64.rpl33	0.01095	6	201
LSC.gene65.rps18	0.00719	6	312
LSC.gene66.rpl20	0.00568	6	387
LSC.gene67.rps12	0.031	22	429
LSC.gene68.clpP	0.02732	45	597
LSC.gene69.psbB	0.00742	31	1527
LSC.gene7.psbK	0.00896	5	186
LSC.gene70.psbT	0	0	139
LSC.gene71.psbN	0.00253	1	141

LSC.gene72.psbH	0.00841	5	240
LSC.gene73.petB	0.00638	11	648
LSC.gene74.petD	0.00835	10	483
LSC.gene75.rpoA	0.01492	41	1014
LSC.gene76.rps11	0.00368	4	417
LSC.gene77.rpl36	0.00292	1	123
LSC.gene78.rps8	0.01481	15	405
LSC.gene79.rpl14	0.01048	11	369
LSC.gene8.psbI	0.00601	2	162
LSC.gene80.rpl16	0.00593	6	405
LSC.gene81.rps3	0.01136	20	657
LSC.gene82.rpl22	0.02507	32	468
LSC.gene83.rps19	0.01147	9	279
LSC.gene9.trnS-GCU	0	0	88
SSC.gene1.ndhF	0.01511	88	2223
SSC.gene10.ndhA	0.01258	38	1092
SSC.gene11.ndhH	0.01359	39	1182
SSC.gene12.rps15	0.01515	10	264
SSC.gene13.ycf1	0.04246	435	5736
SSC.gene2.rpl32	0.01341	6	169
SSC.gene3.trnL-UAG	0.00667	1	80
SSC.gene4.ccsA	0.01663	43	942
SSC.gene5.ndhD	0.01373	51	1521
SSC.gene6.psaC	0.00759	4	246
SSC.gene7.ndhE	0.01394	11	321
SSC.gene8.ndhG	0.01042	14	531
SSC.gene9.ndhI	0.0157	21	504
