# Supplementary material for: Characterization of the Evolutionary Pressure on Anisodus tanguticus Maxim. with Complete Chloroplast Genome Sequence
Source: Genes (Basel). 2022 Nov 15;13(11):2125. doi: 10.3390/genes13112125 (PMC9690199; doi:10.3390/genes13112125)
Supplement: Supplementary file 1 [file genes-13-02125-s001.zip › Table S4.pdf]

Table S4 The Ka/Ks ratios of protein-coding genes of *A. tanguticus* and related species.

Gene	MW246825 vs.KY352315 Ka/Ks	MW246825 vs.NC_030282 Ka/Ks	MW246825 vs.KF248009 Ka/Ks	MW246825 vs.NC_018117 Ka/Ks	MW246825 vs.Z00044.2 Ka/Ks
rps2	0	0	0	0	0
rpoC2	0.421804	1.10328	0.235299	0.359902	0.437101
rpoC1	0.204631	0.102195	0.117028	0.025608	0.057446
rpoB	0.310762	0.227431	0	0.04752	0.125967
petN	NA	-	0	NA	-
psbM	NA	NA	NA	NA	NA
psbD	NA	0	0	0.06301	0
psbC	0.143108	0	0	0.091887	0.081891
psbZ	NA	NA	NA	0	-
rps14	NA	NA	0	0.215481	0.43787
psbA	0	NA	0	0.081657	0
psaB	0	0	0	0.044673	0.090378
psaA	0	0	0	0.049308	0
ycf3	NA	NA	0.284072	0.101773	0.404783
rps4	0.195143	0.196109	0	0	0.066805
ndhJ	0	0.201807	NA	0	0
ndhK	0.442288	0.400158	0.171968	0.265933	0.555537
ndhC	0	0	0	0	0.061499
atpE	0	0	NA	0.769889	0.236214
atpB	0	0	0.114754	0.124406	0.132882
rbcL	0.071828	0.186272	0.173633	0.184738	0.080612
matK	0.461916	0.370966	0.823365	0.421164	0.325497
accD	0.922732	NA	0.674031	0.666601	0.47603
psaI	NA	NA	NA	0.741555	0
ycf4	NA	NA	0	0.435312	0.88838
cemA	1.15724	1.64322	0.373983	0.49684	-
petA	0.110431	0.10281	0.083376	0.161123	0.16937
psbJ	NA	NA	NA	NA	0
psbL	NA	0	NA	NA	NA
psbF	NA	-	NA	NA	NA
psbE	NA	NA	0	NA	0
petL	0	0	0	0	0
rps16	0.706971	0	0.515065	0	0.179076
petG	NA	NA	NA	NA	NA
psaJ	0	0	0	0	0
rpl33	NA	0	0.092898	0	0
rps18	NA	0	NA	0.794666	0
rpl20	0	0	NA	0.207893	0
rps12-2	NA	NA	0.91934	0.673367	-
clpP	1.77581	0.315903	0.088729	1.07052	0.990664

psbB	0.024325	0	0	0	0
psbT	NA	-	NA	NA	NA
psbN	NA	NA	0	NA	NA
psbK	NA	NA	NA	NA	0.290055
psbH	NA	0	NA	0	0
petB	0.253035	0	0.035418	0	0.083467
petD	0.258099	NA	0	0	0
rpoA	0.455228	0.175617	0.276646	0.329624	0.351181
rps11	0	0	0	0	0
rpl36	NA	NA	NA	0	NA
rps8	0.225143	0	0.106281	0.260703	-
rpl14	0	0	0	0.431397	-
rps3	0.863572	0.410814	0.177797	0.225246	-
rpl22	0	0.283013	0.271355	0	-
psbI	0.318592	NA	NA	NA	NA
rps19	0.176059	0.134009	0	0.410053	-
rpl2	NA	0	0	0	-
rpl23-2	NA	NA	NA	NA	-
ycf2-2	NA	0.94969	0.637284	0.934446	-
ycf15-2	NA	NA	NA	NA	-
ndhB-2	NA	NA	NA	0	-
rps7-2	NA	NA	NA	NA	-
rps12-2	NA	NA	0.91934	0.673367	-
ycf1	0.7523		0.489888	0.630553	-
ndhF	0.502659	0.687063	0.195571	0.260442	-
atpA	0.143906	0	0	0.062778	0.086712
rpl32	0.231094	NA	NA	0.32455	-
ccsA	0.179208	0.489156	0.119386	0.15908	-
ndhD	0.122138	0.187358	0.130972	0.128025	-
psaC	0	NA	0	0	-
ndhE	0.21503	0.21503	0	0	-
ndhG	0.622001	0.479522	0.201528	0.156023	-
ndhI	0	0.099831	0.19127	0.098341	-
ndhA	0.254773	0.537279	0.146094	0.451121	-
ndhH	0.132674	0.13348	0.029979	0.021385	-
rps15	0	0	0.148712	0.254474	-
atpF	0.106145	0.766595	0.106145	0.082304	0.383693
ycf1	0.414409	0.48198	0.508044	0.767555	-
rps7-2	NA	NA	NA	NA	-
ndhB-2	NA	NA	NA	0	-
ycf15-2	NA	NA	NA	NA	-
ycf2-2	NA	0.94969	0.637284	0.934446	-
rpl23-2	NA	NA	NA	NA	-
rpl2	NA	0	0	0	-

atpH	0	NA	NA	NA	0.158646
atpI	0.091426	0.09189	0.111249	0.09189	0.075321
