# Supplementary material for: Characterization of the Evolutionary Pressure on Anisodus tanguticus Maxim. with Complete Chloroplast Genome Sequence
Source: Genes (Basel). 2022 Nov 15;13(11):2125. doi: 10.3390/genes13112125 (PMC9690199; doi:10.3390/genes13112125)
Supplement: Supplementary file 1 [file genes-13-02125-s001.zip › Table S5.pdf]

Table S5 The chloroplast genome of plant species for construction phylogenetic tree.

Genbank number	species	Cp genome length
NC_030185.1	Acnistus arborescens	156898 bp
KR078312.1	Capsicum frutescens	156817 bp
NC_018117.1	Datura stramonium	155871 bp
NC_030171.1	Eriolarynx fasciculata	157025 bp
NC_029833.1	Iochroma australe	156996 bp
KF248009.1	Hyoscyamus niger	155720 bp
NC_029833.1	Iochroma Australe	156996 bp
NC_030177.1	Iochroma ellipticum	157052 bp
NC_030168.1	Iochroma salpoanum	156971 bp
NC_007500.1	Nicotiana sylvestris	155941 bp
Z00044.2	Nicotiana tabacum	155943 bp
NC_016068.1	Nicotiana undulata	155863 bp
DQ347958.1	Solanum bulbocastanum cultivar PT29	155371 bp
NC_028070.2	Solanum nigrum	155432 bp
NC_030282.1	Scopolia parviflora	156193 bp
NC_007898.3	Solanum lycopersicum	155461 bp
KP331414.1	Solanum lycopersicum IPA-6	155605 bp
DQ231562.1	Solanum tuberosum	155312 bp
KY352315.1	Przewalskia tangutica	155570 bp
MW246825.1	Anisodus tanguticus	155765 bp
KT428154	Scrophularia dentata	152553 bp
